# Improving the thermostability of a fungal GH11 xylanase via site-directed mutagenesis guided by sequence and structural analysis

**DOI:** 10.1186/s13068-017-0824-y

**Published:** 2017-05-23

**Authors:** Nanyu Han, Huabiao Miao, Junmei Ding, Junjun Li, Yuelin Mu, Junpei Zhou, Zunxi Huang

**Affiliations:** 1 School of Life Sciences, Yunnan Normal University, Kunming, 650500 China; 2 Key Laboratory of Enzyme Engineering, Yunnan Normal University, Kunming, 650500 China

**Keywords:** Xylanase, Thermostability, B-factor, MD simulation, Site-directed mutagenesis, C-terminus replacement

## Abstract

**Background:**

Xylanases have been widely employed in many industrial processes, and thermophilic xylanases are in great demand for meeting the high-temperature requirements of biotechnological treatments. In this work, we aim to improve the thermostability of XynCDBFV, a glycoside hydrolase (GH) family 11 xylanase from the ruminal fungus *Neocallimastix patriciarum*, by site-directed mutagenesis. We report favorable mutations at the C-terminus from B-factor comparison and multiple sequence alignment.

**Results:**

C-terminal residues 207-NGGA-210 in XynCDBFV were discovered to exhibit pronounced flexibility based on comparison of normalized B-factors. Multiple sequence alignment revealed that beneficial residues 207-SSGS-210 are highly conserved in GH11 xylanases. Thus, a recombinant xylanase, Xyn-MUT, was constructed by substituting three residues (N207S, G208S, A210S) at the C-terminus of XynCDBFV. Xyn-MUT exhibited higher thermostability than XynCDBFV at ≥70 °C. Xyn-MUT showed promising improvement in residual activity with a thermal retention of 14% compared to that of XynCDBFV after 1 h incubation at 80 °C; Xyn-MUT maintained around 50% of the maximal activity after incubation at 95 °C for 1 h. Kinetic measurements showed that the recombinant Xyn-MUT had greater kinetic efficiency than XynCDBFV (*K*_m_, 0.22 and 0.59 µM, respectively). Catalytic efficiency values (*k*_cat_ */K*_m_) of Xyn-MUT also increased (1.64-fold) compared to that of XynCDBFV. Molecular dynamics simulations were performed to explore the improved catalytic efficiency and thermostability: (1) the substrate-binding cleft of Xyn-MUT prefers to open to a larger extent to allow substrate access to the active site residues, and (2) hydrogen bond pairs S208-N205 and S210-A55 in Xyn-MUT contribute significantly to the improved thermostability. In addition, three xylanases with single point mutations were tested, and temperature assays verified that the substituted residues S208 and S210 give rise to the improved thermostability.

**Conclusions:**

This is the first report for GH11 recombinant with improved thermostability based on C-terminus replacement. The resulting Xyn-MUT will be an attractive candidate for industrial applications.

**Electronic supplementary material:**

The online version of this article (doi:10.1186/s13068-017-0824-y) contains supplementary material, which is available to authorized users.

## Background

Xylan is the primary hemicellulosic constituent of plant cell walls and accounts for up to 35% of all renewable organic carbon sources on Earth [1]. Xylan is a heterogeneous polysaccharide consisting of a backbone chain of *β*-1,4-d-linked xylose units usually decorated with side groups (such as methyl and acetyl groups) and other sugar molecules [2]. Owing to the heterogeneity and complexity of xylan, complete degradation requires a set of main chain- and side group-cleaving enzymes [3]. Among all the xylanolytic enzymes, endo-*β*-1,4-xylanase (xylanase; EC 3.2.1.8) is a pivotal enzyme that is capable of randomly hydrolyzing the internal *β*-1,4-d-xylosidic linkages in the backbone chain to yield xylooligosaccharides (XOSs) of various chain lengths during xylan bio-degradation [2, 4].

Xylanases have been widely applied in industry, including paper and pulp processing, feed manufacture, and next generation biorefineries [2, 5–7]. Due to the harsh conditions of biotechnological treatments, xylanases with advantageous properties such as excellent thermostability, broad pH adaptability, and high specific activity are in high demand [8]. As such, numerous projects have been undertaken to discover and develop novel xylanases with favorable properties [9–11].

Based on catalytic domain sequence similarity, xylanases from various source organisms are classified into glycoside hydrolase (GH) families 5, 8, 10, 11, 30, and 43 by the CAZy database, and principally belong to GH10 and GH11 [12]. In contrast to GH10 xylanases, GH11 xylanases display higher catalytic efficiency, higher substrate selectivity, and a greater variety of temperature and pH optima [13]. These advantageous properties make GH11 more suited for industrial applications. Thus, the determinants for the improved properties of GH11 have been widely explored [13–17]. Among them, understanding the heat-resistance mechanism for GH11 has become an intense research area owing to the high-temperature requirement in various industrial conditions.

It has been widely accepted that N-terminal region (NTR) of GH11 is of great importance in maintaining xylanase thermostability, and several heat-resistant recombinants have been developed by replacing the NTR with the corresponding parts from thermostable xylanases [17–19]. In 2014, the Rey-Ting Guo group solved the crystal structure of a GH11 xylanase (XynCDBFV) from the ruminal fungus *Neocallimastix patriciarum* [20]. XynCDBFV was identified to have the longest NTR among all GH11 members [20]. The NTR of XynCDBFV folds into an α-helix and tightly attaches to a β-sheet via a disulfide bridge (DS1). It has been shown that the NTR plays an important role in XynCDBFV thermostability and that DS1 is the critical factor joining the NTR to the main body of XynCDBFV [20]. Additionally, one thermophilic xylanase (NFX) from *Nonomuraea flexuosa* has been previously reported and crystallized. This thermophilic NFX was stable at 80 °C and even retained partial activity at 90–100 °C [21]. Although there were no evident structural reasons for the significant higher thermostability of NFX, an additional GNPGNP sequence was found at the C-terminus [21]. Therefore, the C-terminus of NFX and other thermophilic xylanases may also play a pivotal role in maintaining thermostability. A thermophilic xylanase recombinant may be constructed by combining crucial thermostability-enhancing components of xylanases, such as the NTR from XynCDBFV, C-terminus from NFX.

In this work, the sequence and structure of XynCDBFV were carefully compared to those of thermophilic xylanases including NFX. C-terminal residues 207-SSGS-210 were found to be highly conserved in thermophilic xylanases. However, residues 207–210 with sequence NGGA of XynCDBFV were found to display pronounced flexibility. Subsequently, three residues, N207, G208, and A210, from 207-NGGA-210 of XynCDBFV were substituted with serines by site-directed mutagenesis. Experimental measurements showed that the triple mutant displayed higher thermostability and catalytic efficiency than wild-type XynCDBFV. Moreover, heat-resistance mechanisms for the triple mutant were explored by molecular dynamics (MD) simulations and verified by single point mutations. To our knowledge, this is the first report of a GH11 recombinant with improved thermostability based on C-terminus replacement, and the triple mutant demonstrates attractive properties for industrial use.

## Results

### Mutagenesis sites predicted by B-factor comparison

**Fig. 1:**
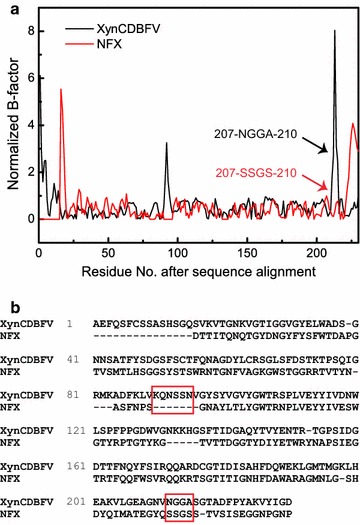
Comparison of normalized B-factors after pairwise sequence alignment. **a** Normalized B-factors were compared between XynCDBFV (*black*) and NFX (*red*). **b** Pairwise sequence alignment between XynCDBFV and NFX

B-factors determined from X-ray diffractions are linearly related to the mean square displacement of atoms relative to their average positions [22]. Therefore, crystal structure B-factors provide useful information about protein dynamics, structural flexibility, and protein stability [23]. In this work, the B-factors of XynCDBFV and NFX were extracted from the crystal structures. After pairwise sequence alignment, normalized B-factors for each Cα atom were compared between XynCDBFV and NFX (Fig. 1a). Two segments corresponding to pronounced flexibility in XynCDBFV were discovered: one segment from residue K86 to residue N91 and another from residue N207 to residue A210. Residues K86 to N91 in XynCDBFV correspond to sequence gaps in NFX (Fig. 1b). Residues N207 to A210 (207-NGGA-210) in XynCDBFV correspond to residues 207-SSGS-210 in NFX and are located at the C-terminal region. It has been suggested that the C-terminus plays a crucial role in maintaining NFX thermostability. Thus, we focused on the second segment of residues N207 to A210.

**Fig. 2:**
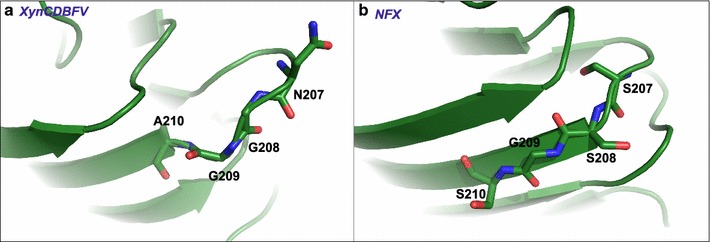
Conformation of residues from 207 to 210 in two xylanases. **a** Conformation of 207-NGGA-210 in the XynCDBFV crystal structure. **b** Conformation of 207-SSGS-210 in the NFX crystal structure

Conformations of residues 207–210 in XynCDBFV and NFX were investigated (Fig. 2). In XynCDBFV, residues 207-NGGA-210 form a β-turn. However, most residues of 207-SSGS-210 in NFX are localized to one β-strand and tightly interact with residues in the anti-parallel β-strand. Moreover, the β-turn linked with 207-SSGS-210 in NFX is more compact than that in XynCDBFV. It is well known that the GH11 xylanase generally folds into a β-jelly-roll structure [8], so the stability of GH11 xylanase is highly related with the arrangement and compactness of β-sheets. Analogous residues 207–210 with different secondary structures may contribute to various stabilities among GH11xylanases.

**Fig. 3:**
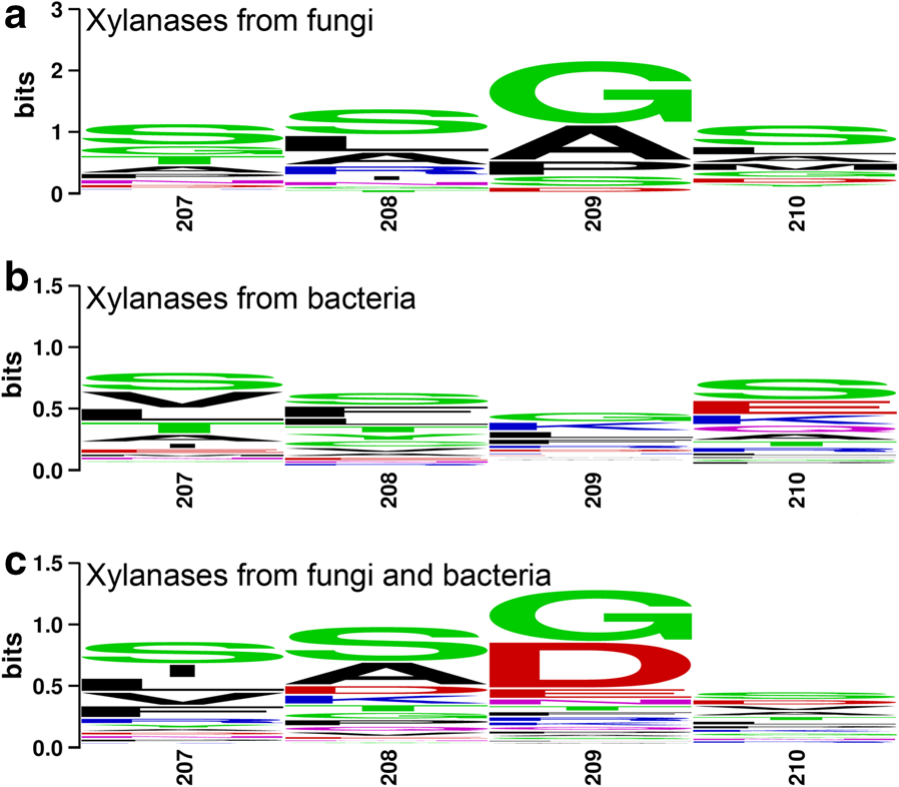
Multiple sequence alignment of GH11 xylanases from different organisms. Sequence logo from multiple sequence alignment from all fungal GH 11 xylanases (**a**), bacterial GH11 xylanases (**b**), fungal and bacterial GH11 xylanases (**c**) from the NCBI protein database

B-factor and structural comparisons indicate that residues 207–210 potentially influence xylanase stability. XynCDBFV is a fungal xylanase from *N. patriciarum* and NFX is a bacterial xylanase from *N. flexuosa*. To investigate sequence diversity of residues 207–210 in different organisms, all GH11 xylanase sequences from fungi and bacteria in the NCBI database were compared (Fig. 3). Multiple sequence alignment revealed that beneficial residues S207, S208, G209, and S210 are highly conserved in both fungal and bacterial GH11 xylanases (Fig. 3a, b). Specifically, residues S207, S208, G209, and S210 account for 31, 31, 48, and 31% in fungal GH11 xylanases, respectively. While the sequence combination of 207-NGGA-210 is much less conserved: N207, G208, G209, and A210 only account for 7, 4, 48, and 11% in fungal GH11 xylanases, respectively. Normalized B-factor comparison, plus multiple sequence alignment suggests 207-SSGS-210 is a putative segment in improving GH11 xylanase thermostability.

### Construction and characterization of Xyn-MUT

**Fig. 4:**
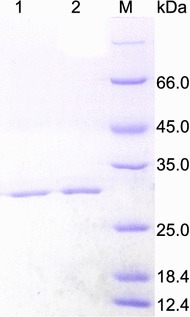
SDS-PAGE analysis of the recombinant xylanases. *Lanes 1* and *2* correspond to purified Xyn-MUT and XynCDBFV from *P. pastoris*, respectively; *lane M* corresponds to standard protein molecular mass markers

Inspired by the B-factor comparison and multiple sequence alignment, residues 207-NGGA-210 in XynCDBFV were substituted to 207-SSGS-210 at equivalent sites by site-directed mutagenesis. The resultant triple mutant (N207S, G208S, A210S) is called Xyn-MUT in this work. Xyn-MUT displayed the same molecular mass (27.37 kDa) as XynCDBFV on SDS-PAGE (Fig. 4). The specific activities of purified Xyn-MUT and XynCDBFV were 920.17 and 798.34 U/mg, respectively.

**Fig. 5:**
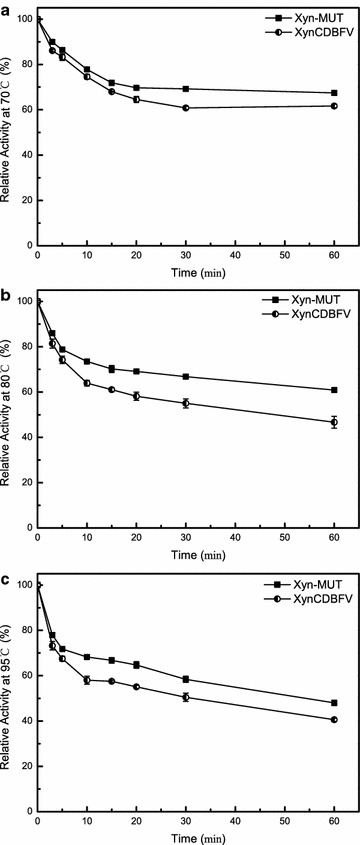
Residual activities of xylanases incubated in different thermal conditions. Residual activities of XynCDBFV and Xyn-MUT expressed in *P. pastoris* incubated at 70 °C (**a**), 80 °C (**b**), and 95 °C (**c**) for 1 h

To evaluate thermostability, residual activities of Xyn-MUT and XynCDBFV were measured after incubation at various temperatures for 1 h. Both Xyn-MUT and XynCDBFV were stable at 70 °C. Residual activities for Xyn-MUT and XynCDBFV were 67 and 62% after 1 h treatment, respectively (Fig. 5a). At temperature >70 °C, Xyn-MUT showed greater residual activity than XynCDBFV. Xyn-MUT retained 61% activity after 1 h incubation at 80 °C, while XynCDBFV retained no greater than 50% activity (47%) at the same condition (Fig. 5b). Moreover, the residual activity for Xyn-MUT was approximately 50% after incubation at 95 °C for 1 h, while the retained activity for XynCDBFV was 40% at the same condition (Fig. 5c). These results highlight that the three substitutions (N207S, G208S, A210S) are advantageous for GH11 xylanase thermostability.

### Kinetic Analysis of Xyn-MUT and XynCDBFV

Kinetic parameters were determined at 37 °C for XynCDBFV and Xyn-MUT. Reactions were monitored using the DNS method at eight concentrations of beechwood xylan, from 0.5 to 5 mg/mL. Kinetic measurements showed that the apparent *K*_m_ values for Xyn-MUT and XynCDBFV were 0.22 and 0.59 μM, respectively (Table 1). The smaller Michaelis constant (*K*_m_) of Xyn-MUT indicates an increase in kinetic efficiency compared to that of XynCDBFV. Moreover, catalytic efficiency (*k*_cat_ */K*_m_) of Xyn-MUT was also increased (1.64-fold). Kinetic analysis revealed that the three substituted residues, although primarily designed to improve thermostability, also enhanced catalytic efficiency and substrate binding.

**Table 1:** Kinetics of Xyn-MUT and XynCDBFV

Enzymes	*V*_max_ (μmol/min/mg)	*K*_m_ (μM)	*K*_cat_ (/s)	*K*_cat_ */K*_m_ (/s/mol)
Xyn-MUT	277.78 ± 2.25	0.22 ± 0.05	125 ± 2.01	568.18 ± 1.98
XynCDBFV	454.55 ± 1.97	0.59 ± 0.08	204 ± 2.23	345.76 ± 2.02

### Stability of mutagenesis sites in MD simulations

To gain insight into the improved thermostability and catalytic efficiency of XynCDBFV, MD simulations for XynCDBFV and Xyn-MUT at different temperatures (65/80 °C) were conducted. The root mean square fluctuation (RMSF) reflects the flexibility for each residue during simulations. Comparing the RMSF values of the mutated residues at 65 and 80 °C in the two xylanases (Table 2), we found that RMSF values of most mutated residues at 80 °C were higher than those at 65 °C in both XynCDBFV and Xyn-MUT, suggesting enhanced flexibility of the mutated residues at elevated temperature. Specifically, mutant residue S207 in Xyn-MUT has smaller RMSF values than N207 in XynCDBFV at both 65 and 80 °C. The Xyn-MUT S208 RMSF (0.1358 nm) was smaller than that of XynCDBFV G208 (0.1524 nm) at 65 °C; however, the situation is reversed at 80 °C, with the Xyn-MUT S208 RMSF (0.1917 nm) larger than that of XynCDBFV G208 (0.1653 nm). The RMSF values of Xyn-MUT S210 and XynCDBFV A210 were almost the same at 65 °C. At 80 °C, the RMSF of Xyn-MUT S210 (0.0769 nm) was smaller than that of XynCDBFV A210 (0.0863 nm). Although the RMSF discrepancies were not as significant as that of the crystallographic B-factors, the smaller RMSF values of Xyn-MUT indicate the mutated sites were relatively stable during simulation.

**Table 2:** RMSF for mutated residues over the whole simulation in XynCDBFV and Xyn-MUT at 65 and 80 **°**C

Enzymes	N/S207 (nm)	G/S208 (nm)	A/S210 (nm)
Xyn-MUT (65 °C)	0.1479	0.1358	0.0789
Xyn-MUT (80 °C)	0.1643	0.1917	0.0769
XynCDBFV (65 °C)	0.1856	0.1524	0.0780
XynCDBFV (80 °C)	0.2052	0.1653	0.0863

### Comparison of the substrate-binding cleft between Xyn-MUT and XynCDBFV

**Fig. 6:**
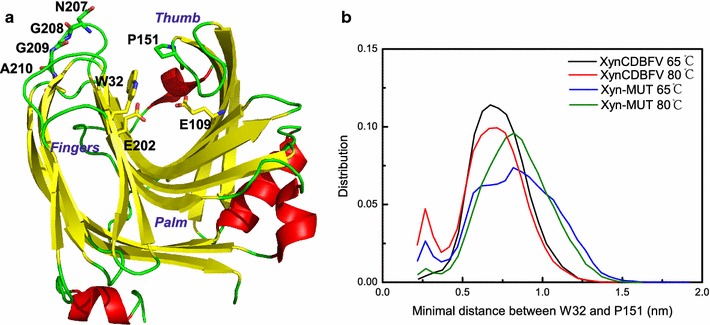
Open and closed states of substrate-binding sites in two xylanases. **a** Schematic structure of XynCDBFV *colored* by secondary structure, β-strand in *yellow*, α-helix in *red* and loop in *green*. **b** Minimal distance distribution between W32 and P151 in XynCDBFV and Xyn-MUT at 65 and 80 °C

The β-jelly-roll catalytic domain of xylanase resembles a partially closed right hand and is made of two anti-parallel β-sheets sculpting a long and deep substrate-binding cleft. The β-sheets form the “palm and fingers” and one long loop forms the “thumb”, which partially closes the cleft. The catalytic residues in XynCDBFV and Xyn-MUT are the same (E109 and E202), and are located at the palm and fingers side of the cleft (Fig. 6a). The thumb loop is highly conserved in GH11 xylanases and is the most flexible region based on crystal structure comparison [24]. It has been suggested that the elevated flexibility of the thumb loop is important to allowing substrate access to the active site. The narrowest zone of the cleft is localized between the conserved proline (P151) in the thumb loop and the conserved tyrosine/tryptophan (W32) in the fingers domain [13]. In order to understand the open and closed states of the substrate-binding cleft in XynCDBFV and Xyn-MUT, the minimal distance between W32 and P151 was calculated each entire simulation trajectory. Comparing the minimal distance (W32–P151) distribution between XynCDBFV and Xyn-MUT (Fig. 6b), it is obvious that the substrate-binding cleft in Xyn-MUT prefers to open to a larger extent at both 65 and 80 °C with the minimal distance centered around 0.8 nm. On the other hand, the minimal distance between W32 and P151 in XynCDBFV at the two temperatures centered at 0.7 nm, indicating a narrower gate to the substrate-binding cleft compared to that of Xyn-MUT. The larger opening of the substrate-binding cleft in Xyn-MUT may contribute to the higher kinetic efficiency of Xyn-MUT.

Additionally, solvent-accessible surface areas for the catalytic residues E109 and E202 were calculated along the whole trajectories (Table 3). The accessible areas of E109 in XynCDBFV and Xyn-MUT were almost the same at both temperatures. Intriguingly, the accessible area of E202 in Xyn-MUT was larger than that of E202 in XynCDBFV at both 65 and 80 °C, indicating that E202 in Xyn-MUT has higher probability of contacting the substrate. The greater W32–P151 minimal distance distribution and greater E202 accessible area of Xyn-MUT versus XynCDBFV provide a molecular-level explanation for the higher kinetic rate and catalytic efficiency.

**Table 3:** Solvent-accessible surface area for catalytic residues (E109 and E202) over the whole simulation in XynCDBFV and Xyn-MUT at 65 and 80 **°**C

Enzymes	E109 (nm^2^)	E202 (nm^2^)
Xyn-MUT (65 °C)	0.25 ± 0.06	0.91 ± 0.09
Xyn-MUT (80 °C)	0.22 ± 0.05	0.91 ± 0.10
XynCDBFV (65 °C)	0.23 ± 0.05	0.87 ± 0.09
XynCDBFV (80 °C)	0.23 ± 0.05	0.88 ± 0.10

### Improved thermostability explored by MD simulations and verified by single point mutations

Generally, mutations that improve thermostability may result via formation of hydrogen bonds, disulfide bridges, stabilization of β-turns or flexible terminuses, enhancement of hydrophobic packing, or α-helix or β-sheet stability [25]. In our work, residues 207–210 were previously discovered to form a β-turn and β-strand in XynCDBFV and NFX crystal structures, respectively (Fig. 2). It is well known that β-strands are connected laterally by hydrogen bonds between backbone chains [26], so we focused on analyzing β-sheet stability and hydrogen bonds interactions with the mutated sites.

**Fig. 7:**
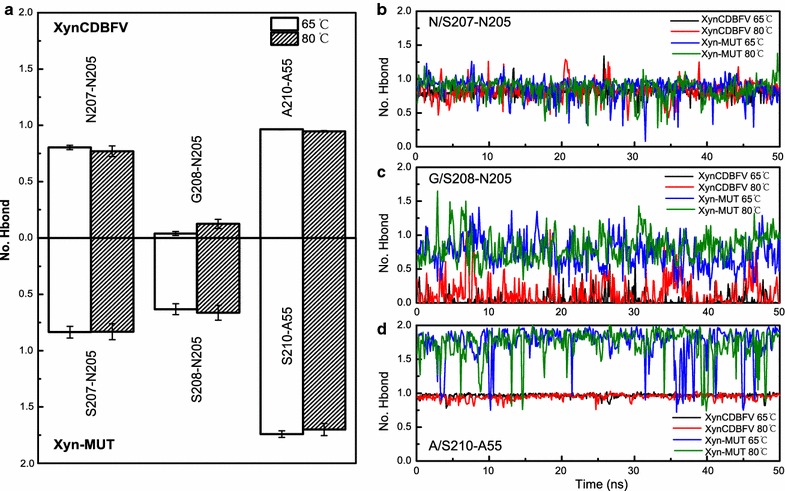
Statistics of hydrogen bond interactions with substituted residues in two xylanases. **a** Number of hydrogen bonds interacted with substituted residues in XynCDBFV (*upper*) and Xyn-MUT (*lower*) over the whole simulations. Number of hydrogen bonds in three pairs N/S207-N205 (**b**), G/S208-N205 (**c**), and A/S210-A55 (**d**) as a function of simulation time in XynCDBFV and Xyn-MUT at 65 and 80 °C

Hydrogen bonds interactions around the C-terminal sites in XynCDBFV and Xyn-MUT were monitored along the whole trajectories (Fig. 7). In XynCDBFV, the probability of hydrogen bonding between N207 and N205 slightly decreased at 80 °C compared to that at 65 °C (Fig. 7a). However, in Xyn-MUT the hydrogen bond connecting S207 and N205 was well preserved at high temperature (80 °C). There was no significant difference in number of hydrogen bonds formed between N/S207 and N205 in XynCDBFV and Xyn-MUT based on time evolution analysis (Fig. 7b). The hydrogen bond connecting S208 and N205 in Xyn-MUT was much stronger (>30%) than that between G208 and N205 in XynCDBFV (Fig. 7a), and the hydrogen bond connecting S208 and N205 in Xyn-MUT was well preserved during the whole simulation (Fig. 7c). S210 and A55 in Xyn-MUT displayed a moderately loose interaction at 80 °C; however, the number of hydrogen bonds between S210 and A55 was 1.85-fold larger than that of XynCDBFV (Fig. 7a). In both XynCDBFV and Xyn-MUT, the backbone nitrogen of A210/S210 forms one stable hydrogen bond with oxygen in the backbone carboxyl group of A55. Moreover, the oxygen (OG) in the side-chain hydroxyl group of S210 in Xyn-MUT has more than 80% probability of forming a hydrogen bond with the oxygen (O) in the backbone carboxyl group of A55 based on structural analysis. The additional hydrogen bond between S210 (OG) and A55 (O) was well maintained during the simulation (Fig. 7d). Thus, the increased number of hydrogen bonds between mutated residue S210 and A55 in Xyn-MUT may be a dominant reason for the improved thermostability. In brief, mutations to S208 and S210 may give rise to the improved thermostability of Xyn-MUT.

**Fig. 8:**
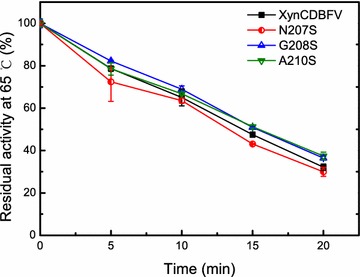
Residual activities of XynCDBFV and single mutants incubated at 65 °C. Residual activities of XynCDBFV and three single mutants (N207S, G208S, and A210S) expressed in *E. coli* incubated at 65 °C for 20 min

Furthermore, the contribution of each individual mutated residue to Xyn-MUT’s thermostability was verified by comparing XynCDBFV to the three single xylanase mutants (N207S, G208S, and A210S) expressed in *Escherichia coli* (Additional file 1: Figure S1). Temperature assays revealed that two single mutants, G208S and A210S, exhibited higher residual activity at 65 °C (Fig. 8). G208S and A210S showed improvements in residual activity with greater thermal retention of 4 and 5%, respectively, compared to XynCDBFV after incubation at 65 °C for 20 min. In contrast, the residual activities of N207S were lower than that of XynCDBFV when incubated at 65 °C, indicating that introducing the N207S substitution did not contribute to higher Xyn-MUT thermostability. The experimental results were in good agreement with the MD simulations, both indicating that the mutated residues S208 and S210 predominantly contribute to the improved thermostability of Xyn-MUT.

**Fig. 9:**
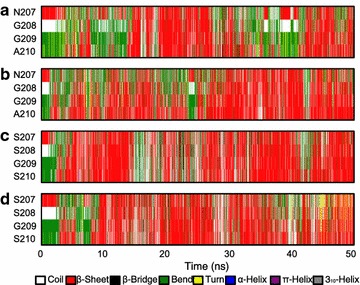
DSSP analysis of two xylanases at different temperatures during the simulations. Analysis of secondary structure propensities of XynCDBFV at 65 °C (**a**) and 80 °C (**b**) during the simulations; analysis of secondary structure propensities of Xyn-MUT at 65 °C (**c**) and 80 °C (**d**)

To decipher structural alterations of the mutated sites during the simulations, secondary structure propensity was analyzed by DSSP [27]. The time evolution of the secondary structure demonstrated the process of conformational conversion (Fig. 9). It is apparent that N207 and G208 in XynCDBFV were associated with a lower propensity for forming a β-sheet than Xyn-MUT (sparse red lines in Fig. 9a, b). Intriguingly, although both XynCDBFV and Xyn-MUT have a glycine at position 209, the propensity of G209 forming a β-sheet was 36% in XynCDBFV at 65 °C (Fig. 9a), but 59% in Xyn-MUT (Fig. 9c). This may be the influence of local secondary structure formation: segment 207-SSGS-210 in Xyn-MUT had a higher propensity for forming a β-sheet than that in XynCDBFV, so G209 in Xyn-MUT’s middle segment would automatically be inclined to take a β-sheet conformation. Moreover, S210 in Xyn-MUT had higher propensity for forming a β-sheet than A210 in XynCDBFV: S210 had 57 and 50% propensities to form a β-sheet in Xyn-MUT at 65 and 80 °C, respectively (Fig. 9c, d); however, A210 had no more than 50% propensity for forming a β-sheet in XynCDBFV at both temperatures (Fig. 9a, b). Above all, secondary structure propensity analysis revealed that residues in 207-SSGS-210 of Xyn-MUT had a higher propensity for forming a β-sheet structure than 207-NGGA-210 in XynCDBFV.

## Discussion

Developing a thermostable xylanase is of great value to meeting practical industrial demands. Additionally, enhanced temperature stability may also improve kinetic efficiency [28]. In this work, B-factor comparison and multiple sequence alignment were first performed to guide the design of mutations to obtain a thermostable xylanase. The result, Xyn-MUT, constructed by site-directed mutagenesis, showed higher thermostability and catalytic efficiency, and the heat-resistance mechanisms of Xyn-MUT were explored by MD simulations and single point mutations.

Firstly, the comparison of normalized B-factors between XynCDBFV and thermophilic NFX is the most crucial step in constructing a thermostable recombinant in this work. Systematic structural studies regarding various enzymes have demonstrated that thermophilic enzymes are characterized by higher degrees of rigidity [29]. Therefore, a strategy for enhancing the thermostability of a particular enzyme is to increase its rigidity at appropriate sites. The B-factors from X-ray data provide information on the fluctuation, and hence, rigidity of atoms relative to their equilibrium positions [22]. Previous work has successfully demonstrated improving enzyme thermostability through the interpretation of B-factors [30]. Consequently, we calculated and compared normalized B-factor values from XynCDBFV and NFX, determined residues with pronounced degrees of flexibility, and constructed a recombinant Xyn-MUT by mutating the flexible residues. Temperature stability testing and kinetic analysis revealed that Xyn-MUT displays higher thermostability and catalytic efficiency than XynCDBFV. Experimental measurements validate the accuracy of B-factor interpretation.

Secondly, Serine (Ser) at the C-terminus is highly related to thermostability in GH11 xylanase. Multiple sequence alignment showed that residues S207, S208, and S210 are highly conserved in both fungal and bacterial GH11 xylanases. The recombinant Xyn-MUT was produced by substituting residues in position 207, 208, and 210 to Ser. The more thermostable Xyn-MUT indicates that incorporating Ser is a potent strategy in engineering a thermostable GH11 xylanase. In another study, Ser- and Thr-containing mutants displayed less flexibility in thermophiles than in mesophiles based on analysis of B-factors from mesophilic and thermophilic proteins [23], suggesting that Ser and Thr are associated with high rigidity in thermophiles. Accordingly, this approach to engineering a thermostable enzyme requires two steps: (1) determine appropriate sites with high flexibility; and (2) mutate flexible sites to appropriate amino acids that allow high rigidity. In our work, the first step was assisted by B-factor comparison. In the second step, the flexible residues were mutated to Ser, a residue that contributes to high rigidity in thermophiles.

Thirdly, this is the first report of a GH11 recombinant with improved thermostability based on C-terminal replacements. Until now, rational design of improved GH11 xylanases have typically focused on three aspects: (1) replacement of the NTR with corresponding parts from thermostable enzymes [17–19]; (2) stabilization of α-helices by introducing disulfide bridges or electrostatic interactions [21, 31]; (3) modification of surface characteristics to form a tighter packing enzyme with fewer cavities [16, 32]. In this work, we found a dominant sequence 207-SSGS-210 at the C-terminus and replaced 207-NGGA-210 in XynCDBFV with this dominant sequence. The thermostable recombinant provides new opportunities for engineering GH11 xylanases at the C-terminal region.

**Fig. 10:**
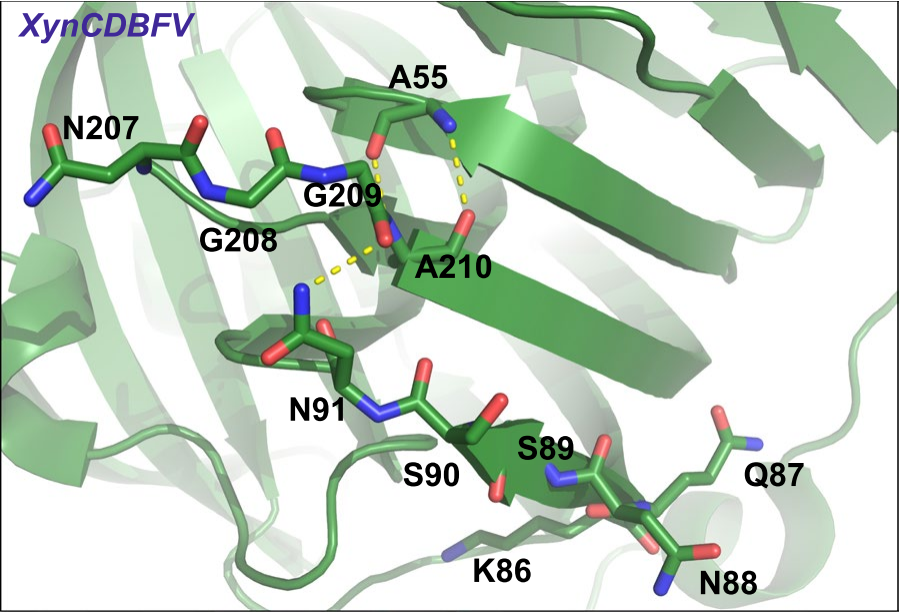
Conformations of 207-NGGA-210 and 86-KQNSSN-91 in XynCDBFV crystal structure

Fourthly, the first segment 86-KQNSSN-91 determined by B-factor comparison may also influence the stability of XynCDBFV. This segment corresponds to sequence gaps in NFX from pairwise sequence alignment. We did not construct XynCDBFV mutants that delete residues 86-KQNSSN-91 in this work. However, the residues 86-KQNSSN-91 are located adjacent to the β-strand formed by the second segment 207-NGGA-210 (Fig. 10). Moreover, N91 interacts with A210 through a hydrogen bond. Residues 207-NGGA-210 displayed flexibility during MD simulations; the N91 interaction plus other residues in 86-KQNSSN-91 may also disturb the stability of XynCDBFV. This conjecture requires experimental studies.

Fifthly, the additional GNPGNP sequence at the C-terminus of NFX is associated with larger-than-average normalized B-factor values (Fig. 1a), indicating it may serve as a flexible linker at the C-terminus. This may allow relatively free movement of the C-terminal domain. Although the GNPGNP sequence from NFX may not contribute to thermostability, it may assist in maintaining NFX’s function.

Lastly and importantly, XynCDBFV and its mutants expressed in *E. coli* are less active than that expressed in *Pichia pastoris*. The *E. coli* expression system was used to test the thermostability contribution of individual mutated residues in isolation, since the *E. coli* expression system is easier to handle than the *P. pastoris* expression system. However, we discovered that XynCDBFV and its three single mutants expressed in *E. coli* exhibit their highest activities at no more than 65 °C. Similar phenomena have been observed in several studies [20, 33, 34], indicating that *P. pastoris* is a better production host than *E. coli* for XynCDBFV expression and performance, and a more suitable expression system for commercial applications. Nevertheless, the enhanced thermostability of Xyn-MUT accounted for by replacing G208 and A210 in XynCDBFV with serines.

## Conclusions

In this work, we identified for the first time the conserved C-terminal residues 207-SSGS-210 of GH11 xylanases and constructed a recombinant xylanase, Xyn-MUT. Experiments showed Xyn-MUT had higher thermostability and kinetic and catalytic efficiency than those of its template (XynCDBFV). Heat-resistance mechanisms explored by MD simulations and single point mutation studies revealed that S208 and S210 give rise to this improved thermostability. Additionally, residues 207-SSGS-210 assist Xyn-MUT in forming a more stable and compact β-sheet structure. The resulting Xyn-MUT is an attractive candidate in industrial applications. This work confirms that sequence/structural-guided protein engineering is an effective strategy for obtaining a thermostable enzyme.

## Methods

### Materials

High-fidelity DNA polymerase, restriction endonuclease (*Nde*I, *Xho*I), and dNTP were purchased from TaKaRa (Otsu, Japan). pEASY-E2 expression vector and pMD19-T vector were purchased from TaKaRa (Otsu, Japan). pPICZA expression vector was purchased from Invitrogen (Shanghai, China). The plasmid mini-prep kit and DNA gel extraction kit were purchased from Omega (Taipei, USA). One Step cloning kit was purchased from Vazyme biotech (Nanjing, China). Fast MultiSite Mutagenesis System and Bradford protein assay kit were purchased from TransGen (Beijing, China). *Escherichia coli* Trans1-T1 cells, *E. coli* BL21 (DE3) cells, and *E. coli* DMT cells were purchased from TransGen (Beijing, China). All other chemicals were of analytical grade and commercially available.

### Gene cloning and site-directed mutagenesis

*XynCDBFV* (GenBank:KP691331) was synthesized in Generay (Shanghai Generay Biotech Co. Ltd.). The gene of xylanase triple mutant (*Xyn*-*MUT*) and three xylanase single mutants (*N207S*, *G208S*, and *A210S*) were constructed by introducing mutations to *XynCDBFV* through site-directed mutagenesis. Mutations causing the amino acid exchanges were introduced using the Fast MultiSite Mutagenesis System according to the manufacturer’s instructions. Forward and reverse primers for *XynCDBFV*, *Xyn*-*MUT*, and three single mutant genes are listed in Table 4. The *XynCDBFV* and *Xyn*-*MUT* PCR products were linked with pPICZA vector and then transformed into *P. pastoris* system; the resulting recombinants containing *XynCDBFV* and *Xyn*-*MUT* were confirmed by DNA sequencing. On the other hand, *N207S*, *G208S*, *A210S*, and *XynCDBFV* PCR products were linked with pEASY-E2 vector and then transformed into *E. coli* Trans1-T1 for sequencing. PCR cycling conditions consisted of an initial step of 5 min at 94 °C, followed by 30 cycles of 30 s at 94 °C, 1 min at 55 °C, and 3 min 30 s at 72 °C.

**Table 4:** Oligonucleotide primers for *XynCDBFV*, *Xyn*-*MUT*, and three single mutant genes

Primers	Primer sequences
XynCDBFV-forward	5′-CAAAGTTTCTGTAGTTCAGCTTCTC-3′
XynCDBFV-reverse	5′-ACCAATGTAAACCTTTGCGTATG-3′
Xyn-MUT-1-forward	5′-GGTGAAGCCGGTAACGTTA**G**C**A**GTGGTGCCAGTG-3′
Xyn-MUT-1-reverse	5′-CAC**T**G**C**TAACGTTACCGGCTTCACCTAAAACCTTG-3′
Xyn-MUT-2-forward	5′-GGTAACGTTAGCAGTGGT**T**CCAGTGGTAC-3′
Xyn-MUT-2-reverse	5′- **A**ACCACTGCTAACGTTACCGGCTTCACCTAAAAC-3′
N207S-forward	5′-TGAAGCCGGTAACGTTA**G**CGGTGGTGCCAGT-3′
N207S-reverse	5′-**C**TAACGTTACCGGCTTCACCTAAAACCTTG-3′
G208S-forward	5′-GAAGCCGGTAACGTTAAC**A**GTGGTGCCAGT-3′
G208S-reverse	5′-**T**GTTAACGTTACCGGCTTCACCTAAAACCT-3′
A210S-forward	5′-GGTAACGTTAACGGTGGT**T**CCAGTGGTACCG-3′
A210S-reverse	5′-**A**ACCACCGTTAACGTTACCGGCTTCACCTA-3′

Nucleotide resulting in the desired mutation is underlined and in bold

### Protein expression and purification

Plasmids pPICZA/*XynCDBFV* and pPICZA*/Xyn*-*MUT* were linearized by PmeI and then individually transformed into *P. pastoris* GS115 by electroporation. The transformants were selected on YPD (yeast extract peptone dextrose) plates containing 200 μg/mL zeocin. The selected clones were inoculated and amplified in 30 mL of buffered glycerol-complex medium at 30 °C for 2 days. Then the culture medium was replaced by 20 mL of buffered methanol-complex medium to induce protein expression. For purification, the supernatants were concentrated using Amicon centrifugal filter device (cutoff 3.000). On the other hand, *E. coli* BL21 (DE3), harboring pEASY-E2 vector that link *XynCDBFV* and three single mutant genes, was grown overnight at 37 °C in Luria–Bertani (LB) medium supplemented with 100 μg/mL ampicillin. Afterwards, the culture was transferred into fresh LB medium containing 100 μg/mL ampicillin by 1% dilution at 37 °C. IPTG (0.1 mM) was added until cell density (OD_600nm_) reached 0.6–0.8, then the culture was grown at 22 °C for 12 h [35]. Cells were then harvested by centrifugation and resuspended with PBS buffer (pH 7.0, 140.0 mM NaCl, 2.7 mM KCl, 10.0 mM Na_2_HPO_4_, 1.8 mM KH_2_PO_4_). Supernatant was collected after the cells were disrupted by sonication and centrifugation at 15,000 rpm for 30 min at 4 °C. The C-terminal His-tagged xylanases were purified using a column of Ni–NTA agarose. The success of the purification was determined by SDS-PAGE, and enzyme concentration was determined by Bradford protein assay kit.

### Enzyme activity characterization

Xylanase activity was analyzed by measuring the release of reducing sugars (xylose) liberated from birchwood xylan using 3,5-dinitrosalicylic acid under optimum conditions [36]. One activity unit (1 U) was defined as the amount of xylanase required to liberate 1 μmol of reducing sugars per minute. All assays in this work were performed in triplicate. Thermostability was assayed by measuring residual enzyme activity after incubation at 70, 80, and 95 °C for 1 h under xylanase optimal pH. The residual enzyme activities were measured under standard assay.

The kinetic parameters (*K*_m_, *V*_max_, and *k*_cat_) for purified xylanases were determined in McIlvaine buffer (pH 5.5) at 37 °C. Birchwood xylan was used as the substrate. The reactions were monitored using the DNS method at eight concentrations of beechwood xylan, from 0.5–5 mg/mL. The kinetic values were calculated by fitting the Lineweaver–Burk plot [37].

### Prediction of the mutagenesis sites by B-factor comparison

To evaluate the main chain flexibility, B-factors of the Cα atoms in XynCDBFV and NFX were extracted from the PDB files [20, 21]. Because the B-factors in different PDB files were refined in different procedures, they cannot be directly compared [38]. Thus, the B-factors in each protein were normalized to have a distribution of zero mean and unit variance based on the following equation:

$$B^{\prime} = \frac{B - \langle B\rangle }{\sigma (B)},$$

where the 〈*B〉* is the average of all Cα atoms and *σ*(*B*) is the standard deviation of the B-factors for the individual protein [39]. The above equation has already been testified and used by other groups [40, 41].

### MD simulation details

The X-ray crystal structure of XynCDBFV was taken from PDB 3WP4 [20] and the recombinant with three point mutations (N207S, G208S, A210S), Xyn-MUT, was built by SWISS-MODEL sever [42]. Normal MD simulations of XynCDBFV and Xyn-MUT were performed at 65 and 80 °C. After 1000-step energy minimization, all the systems were first equilibrated for 5 ns in NPT ensemble followed by another 5 ns equilibration in NVT ensemble by restraining all heavy atoms. Finally, each system was simulated for 50 ns, and the simulation time for all the simulation systems was 200 ns in total. All systems were solvated with TIP3P waters in an octahedral box [43], and the minimal distance between each protein and edge of the box was set to 0.8 nm. Sodium and chloride ions were added with a concentration of 100 mM to neutralize the systems. Protonation states for histidines were determined by the UCSF Chimera program [44]. The GROMACS program suite version 4.5.7 and Amber ff99SB force field were used in all simulations [45, 46]. The simulations were performed in an isothermal–isobaric ensemble (65/80 °C, 1 bar). Bond length constraints were applied to all bonds that contained hydrogen atoms based on the LINCS protocol [47]. An integration step of 0.002 ps was used in all simulations. Electrostatic integrations were treated with Particle Mesh Ewald method with a cutoff of 0.9 nm with grid spacing for the FFT grid <0.12 nm [48].

### Hydrogen bond analysis

Hydrogen bonds between mutational residues and nearby residues in all simulation systems were analyzed by using g_hbond in the GROMACS suite. Geometrical criterions which include donor–acceptor distance and hydrogen-donor–acceptor angle are used to calculate hydrogen bond. In Fig. 7a, the number of hydrogen bonds was calculated based on the whole 50 ns simulation in each system, and the error bar represents one standard error which was calculated based on the averaged number of hydrogen bonds every 10 ns in each system. In Fig. 7b–d, the hydrogen bond forming probability was analyzed every 100 ps during the whole simulation.

## Additional file

**Additional file 1: Figure S1.** SDS-PAGE analysis of the recombinant xylanases. Lanes 1, 3, 5 and 7 correspond to purified XynCDBFV, N207A, G208S, and A210S from *E. coli* BL21 (DE3), respectively; lanes 2, 4, 6 and 8 correspond to expressed XynCDBFV, N207A, G208S, and A210S, respectively; lane 9 corresponds to control cell (harboring empty pEasy-E2 vector); lane M corresponds to standard protein molecular mass markers.

## Abbreviations

DS1: disulfide bridge
*E. coli*: *Escherichia coli*
GH: glycoside hydrolase
LB: Luria–Bertani
MD: molecular dynamics
NTR: N-terminal region
*P. pastoris*: *Pichia pastoris*
XOSs: xylooligosaccharides
