# Supplementary figures and images for: Improving the thermostability of a fungal GH11 xylanase via site-directed mutagenesis guided by sequence and structural analysis
Source: Biotechnol Biofuels. 2017 May 23;10:133. doi: 10.1186/s13068-017-0824-y (PMC5442702; doi:10.1186/s13068-017-0824-y)

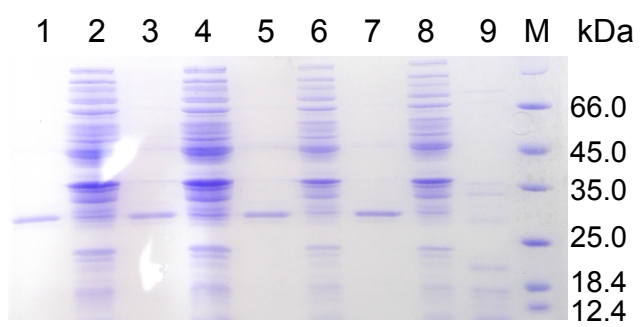

Supplement: Supplementary file 1 — Additional file 1: Figure S1. SDS-PAGE analysis of the recombinant xylanases. Lanes 1, 3, 5 and 7 correspond to purified XynCDBFV, N207A, G208S, and A210S from E. coli BL21 (DE3), respectively; lanes 2, 4, 6 and 8 correspond to expressed XynCDBFV, N207A, G208S, and A210S, respectively; lane 9 corresponds to control cell (harboring empty pEasy-E2 vector); lane M corresponds to standard protein molecular mass markers. [file 13068_2017_824_MOESM1_ESM.pdf]
